# Toxicity and Metabolomic Dysfunction Invoked by Febrifugin, a Harmful Component of Edible Nut of *Swietenia macrophylla*

**DOI:** 10.3390/ijms25179753

**Published:** 2024-09-09

**Authors:** Xiaoyue Zhang, Qinyang Song, Hanghang Zheng, Rui Wang, Qiang Zhang

**Affiliations:** 1 Shaanxi Key Laboratory of Natural Products & Chemical Biology, College of Chemistry & Pharmacy, Northwest A&F University, Yangling 712100, China; xiaoyue.zhang@nwafu.edu.cn (X.Z.); 2022051653@nwafu.edu.cn (Q.S.); 2 National Narcotics Laboratory Shaanxi Regional Center, Narcotics Technical Center of Shaanxi Provincial Public Security Department, Key Laboratory of Drugs Analysis & Intelligent-Monitoring, Xi’an 710115, China; zhh18710683097@163.com (H.Z.); 18487165159@163.com (R.W.)

**Keywords:** *Swietenia macrophylla*, febrifugin, toxicity, metabolism, pentose phosphate pathway, TCA cycle

## Abstract

*Swietenia macrophylla* fruit is a valuable and historically significant medicinal plant with anti-hypertension and anti-diabetes. We identified a toxic component, Febrifugin, from the edible part of the nut following zebrafish toxicity-guided isolation. Febrifugin is a mexicanolide-type limonoid compound. The toxic factor induced acute toxicity in zebrafish, including yolk sac edema and pericardial edema, reduced body length, decreased melanin deposition, and presented acute skeletal developmental issues. Further exploration of the acute toxicity mechanism through metabolomics revealed that Febrifugin caused significant changes in 13 metabolites in zebrafish larvae, which are involved in the pentose phosphate, tricarboxylic acid (TCA) cycle, and amino acid biosynthesis. The bioassay of oxidative stress capacity and qRT-PCR measurement showed that the compound significantly affected the *h6pd* gene in the pentose phosphate pathway and the mRNA expression of *cs*, *idh3a*, *fh*, and *shda* genes in the TCA cycle, leading to reactive oxygen species (ROS) accumulation and a notable decrease in glutathione (GSH) activity in zebrafish. These findings provide a basis for the rational use of *S. macrophylla* as a medicinal plant and raise awareness of the safety of medicinal plants.

## 1. Introduction

Food safety has consistently been a major public health issue in modern society. Given the extensive history of medicinal plant use, consumers often assume these plants are safe for consumption. While plants are naturally derived and typically regarded as healthy and beneficial, some medicinal plants may contain substances that can be harmful to human health. These harmful substances can include natural toxins such as cytotoxic quinolone alkaloids found in the fruits of *Evodia rutaecarpa* [1]. Our research group has also identified toxic components, such as Lansamide I from *Clausena lansium* fruits [2] and Esculentoside B from *Phytolacca acinosa*, a plant used medicinally and for food [3].

Focusing on harmful substances in fruits is important for fruit safety and human health. The toxic elements in fruits can pose health risks when consumed excessively and accumulate in the human body, leading to various health issues like neurological damage, anemia, and an increased risk of cancer [4]. Therefore, understanding and monitoring harmful substances is vital to food safety, protecting consumers from health risks, ensuring compliance with safety standards, identifying contamination sources, and empowering consumers to make informed choices.

*Swietenia macrophylla*, known as mahogany, has been studied for its diverse chemical constituents isolated from different plant parts. The plant harbors diverse chemical constituents with distinct structural features and biological activities. The main compounds found in *S. macrophylla* include limonoids, polyacetylenes, triterpenoids, phenolic compounds, and essential oils, each showing unique structural characteristics and biological activities. Limonoids are a prominent group of compounds found in various plant parts, including seeds, bark, and leaves [5,6]. These limonoids from *S. macrophylla* have shown inhibitory activity against apoptosis [7], potential antitumor activities in human malignant melanoma cells [8], and moderate cytotoxic effects against cancer cell lines. Moreover, these compounds have demonstrated antioxidative activities and larvicidal properties [9]. Triterpenoids in the plant fruit demonstrate cytotoxic activities. Phenolic compounds in the plant, such as xanthones and essential oils, showed potential antioxidant and anti-inflammatory properties, which are valuable for various applications, including skin care [10]. Limonoids have been reported to possess antifeedant and insecticidal properties towards insects or toxicity against cancer cells. Azadirone-, nimbin-, and salannin-type limonoids generally exhibit potent or moderate cytotoxic activities. Among these, the azadirone- and salannin types are more active than the nimbin-type limonoids [11]. These findings also suggest that certain types of limonoids can be potentially toxic substances.

Here, we report a toxic limonin found in *S. macrophylla* and comprehensively evaluate its effects on embryonic development and in vivo metabolism, which provides a scientific basis for the rational use of medicinal plants. Additionally, this study emphasizes the necessity of avoiding excessive intake of medicinal plants to prevent potential toxic side effects, thereby ensuring public health and safety.

## 2. Results

### 2.1. Discovery of Toxic Factors in Swietenia Macrophylla and Its Chemical Constituents

**Figure 1:**
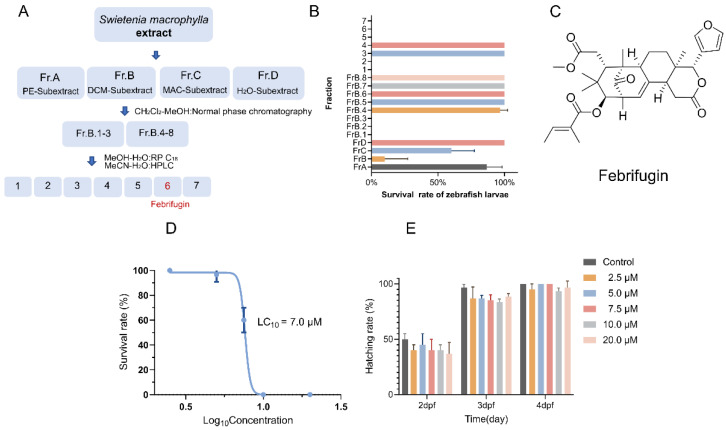
(**A**) Separation process of toxic components in *S. macrophylla*. (**B**) Biologically guided separation evaluation. (**C**) Chemical structure of the main toxic compound Febrifugin. (**D**) Concentrations lead to differences in survival rates. (**E**) Incubation success rate (*n* = 3, with 20 larvae in each replicate).

*S. macrophylla* is an important medicinal plant from which several limonoid triterpenoids with cytotoxic effects have been isolated. Current medical records indicate that the amount of seeds people typically consume per day is strictly controlled, which has inspired our interest. In this study, we used zebrafish larvae for bioactivity-guided separation to assess the toxicity of various components (Figure 1A).

As shown in Figure 1B, after extraction with solvents of different polarities and measurement at a uniform time point, the mortality rate of 3 dpfs (days post fertilization) zebrafish larvae exposed to FrB was 90%, significantly higher than other components (specific components are mentioned). We performed further separation of this component using normal-phase chromatography, resulting in eight fractions. We found that FrB.1-3 had a mortality rate of 100% under exposure. We then purified FrB.1-3 through multiple HPLC steps and obtained a compound that caused the fastest complete death of zebrafish within the same time frame, identified by ^1^H, ^13^C, and LC-MS/MS as Febrifugin (Figure 1C). Then, we analyzed the content of Febrifugin in the nut of *S. macrophylla*, using [M+H]^+^ peak with *m*/*z* 553.2787 and a retention time of 17.19 min in LC-MS/MS. The contents of Febrifugin in the peel and the kernel were 0.0053% and 0.0064%, respectively, according to the area normalization method for content analysis.

To further evaluate the toxicity of Febrifugin and explore its mechanism of action, as shown in Figure 1D, when the exposure concentration exceeded 5.0 μM, zebrafish larvae began to show signs of death, and at concentrations above 7.5 μM, noticeable death symptoms appeared. Therefore, the LC_10_ concentration of Febrifugin is 7.0 μM. Subsequently, we statistically analyzed the hatching of zebrafish larvae exposed to Febrifugin (Figure 1E). We observed that within the statistical time range, Febrifugin did not affect normal zebrafish hatching. The hatching rate at a concentration of 20.0 μM was also 96.67%, which was inconsistent with the acute toxicity results. Therefore, for the subsequent acute toxicity mechanism, we chose concentrations (4.0 μM and 6.5 μM) lower than LC_10_ because this concentration level ensured sufficient survival of zebrafish in the experiment, allowing for the observation of sublethal effects, which also helped evaluate the toxic effects of the test substance [12].

### 2.2. Developmental Toxicity of Febrifugin

**Figure 2:**
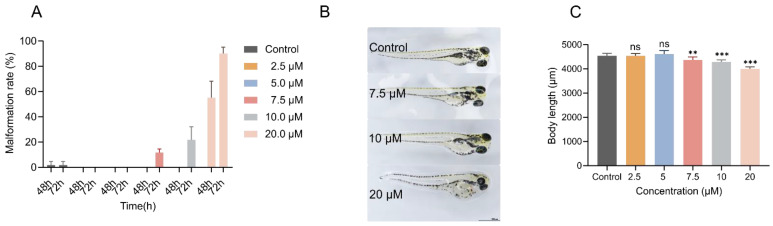
(**A**,**B**) Abnormality occurrence rate (*n* = 3, 20 larvae per replicate). (**C**) Length measurement of zebrafish at the developmental stage of 5 dpf, (*n* = 10). ImageJ (Version 1.54) was used for evaluating the body length of zebrafish larvae, bar = 1000 μm (ns, not significant; **, *p* < 0.01; ***, *p* < 0.001).

Although Febrifugin did not affect normal zebrafish hatching, its developmental toxicity still needs further evaluation. First, we assessed the deformity rate of larvae at 48h and 72 hpf (hours post fertilization). The deformity rate was evaluated by observing the morphological changes in zebrafish larvae under a microscope after treatment with different drug concentrations, checking for pericardial edema and yolk sac enlargement. As shown in Figure 2A, after 48 h of treatment, only the 20.0 μM treatment group showed morphological changes in zebrafish, with a deformity rate of 55.0%. After 72 h, zebrafish in the 7.5 μM and 10.0 μM treatment groups showed morphological changes, with 90.0% of the 20.0 μM treatment group displaying deformities.

Simultaneously, we quantitatively analyzed the body length of zebrafish larvae. As shown in Figure 2B,C, we found that larvae (4 dpf) treated with Febrifugin concentrations above 7.5 μM had significantly reduced body length, exhibiting significant dilation of the yolk sac and swelling around the heart cavity affecting development. However, at 2.5 μM and 5 μM treatments, the body length was similar to the control group.

**Figure 3:**
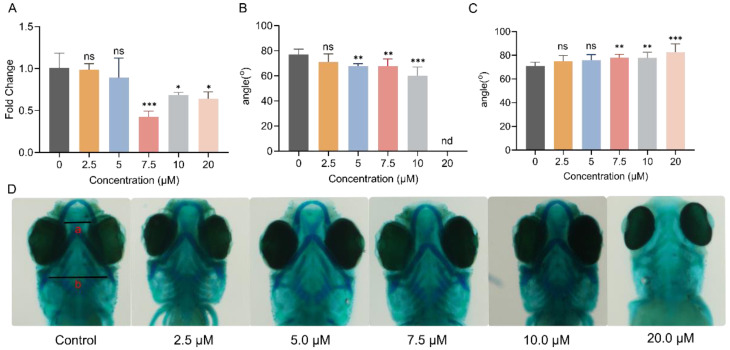
(**A**) Melanin content (*n* = 3; ns, not significant; *, *p* < 0.05; **, *p* < 0.01; ***, *p* < 0.001), the melanin content of the blank group was used for normalization, calculating the fold change. (**B**) Skeletal development, Meckel’s cartilage angle; nd, not detected, and not included in statistical analysis. (**C**) Ceratohyal bone angles (*n* = 10). (**D**) Image of a zebrafish head stained with Alcian blue; a, Meckel’s cartilage angle; b, ceratohyal bone angles.

As part of evaluating the impact of Febrifugin on zebrafish morphology, another interesting factor is pigment deposition. Our data indicate that Febrifugin exposure led to reduced melanin accumulation in zebrafish (Figure 3A). At 7.5 μM, there was a significant difference, with melanin accumulation reduced by 58.7% compared to the control group. At 10.0 μM and 20.0 μM, the reductions were 32.6% and 36.0%, respectively. To determine the impact of Febrifugin on zebrafish cartilage development, Alcian blue staining was performed at 72 hpf (Figure 3B–D). The Alcian blue staining results showed abnormal development in the craniofacial skeletal structures of zebrafish, mainly characterized by an increased angle between the ceratohyal bones (CH angle). After treatment with concentrations greater than 7.5 μM, the angles increased by 9.0%, 9.7%, and 13.9%, respectively, showing a significant difference compared to the control group. The progression of the Meckel’s cartilage angle, denoted as the MA angle, experienced a marked impact, demonstrating a consistent decline across various treatment levels in contrast to the control group. Particularly at 20.0 μM, some larvae exhibited severe Meckel’s cartilage loss, making quantification impossible. This indicates severe abnormalities in cartilage development in zebrafish larvae. Although normal hatching was not affected, different concentrations of Febrifugin exposure resulted in varying degrees of growth inhibition.

### 2.3. Metabolic Profile Changes in Zebrafish under Febrifugin Exposure

**Figure 4:**
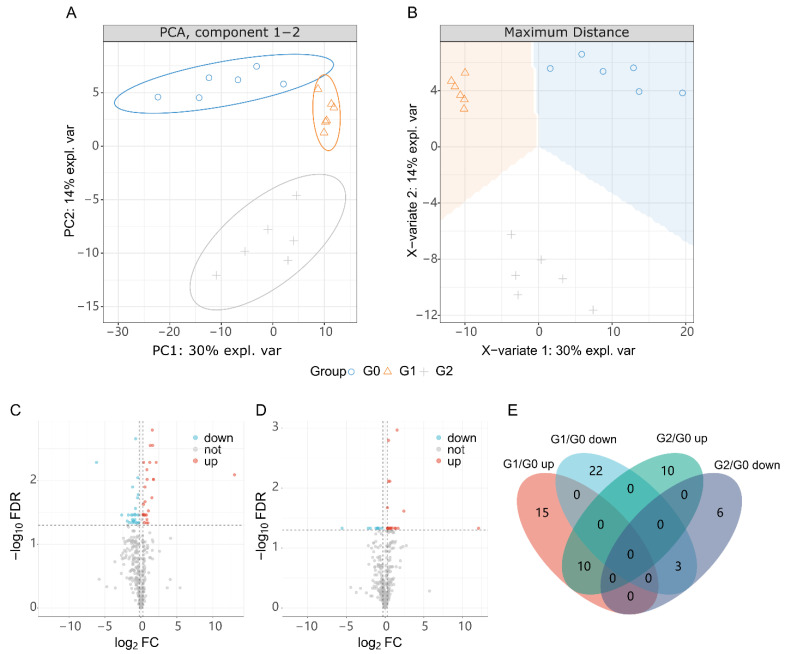
Metabolic profile changes in zebrafish under Febrifugin exposure. Comparative PCA (**A**) and sPLS-Da (**B**) LC-MS/MS metabolic data. Volcano plots of G1 vs. G0 (**C**) and G2 vs. G0 (**D**). (**E**) Number of changed (differential) metabolites in groups. FDR, *p* values corrected by the method false discovery rate; FC, fold change. G0, blank control; G1 and G2, treated with 4.0 μM and 6.5 μM Febrifugin respectively.

We investigated the metabolic characteristics of zebrafish larvae exposed to Febrifugin at concentrations below LC_10_ (G1, 4.0 μM and G2, 6.5 μM). Combining statistically corrected *p*-values and fold changes (FCs), we identified a total of 344 metabolites that changed in the control group and under 4.0 μM, 6.5 μM exposure (Figure 4). Specifically, 50 differential metabolites (DMs, 25 up-regulated and 25 down-regulated) were found at the 4.0 μM treatment. At 6.5 μM, 29 metabolites showed differential changes (20 up-regulated and 9 down-regulated).

**Figure 5:**
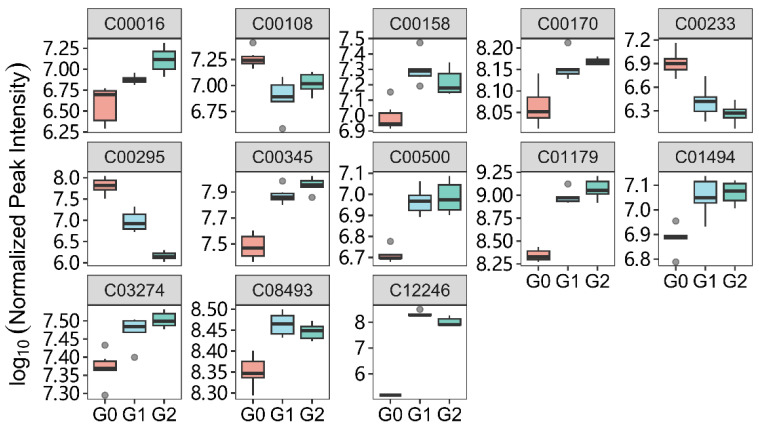
Differential metabolite change levels. C00016, FAD; C00108, Anthranilic acid; C00158, citrate; C00170, 5′-Methylthioadenosine; C00233, 4-Methyl-2-oxopentanoate; C00295, Orotate; C00345, 6-Phospho-D-gluconate; C00500, biliverdin; C01179, 3-(4-Hydroxyphenyl)pyruvate; C01494, Ferulate; C03274, Glycerophosphoglycerol; C08493, Indole-3-carboxaldehyde; and C12246, Protorifamycin I. Peak intensity came from the extract chromatography of LC-MS/MS analysis and normalized by quanlity control and internal standard. G0, blank control; G1 and G2, treated with 4.0 μM and 6.5 μM Febrifugin respectively.

To study the associated effects at different concentrations, we focused on metabolites that showed significant differences in both G1 and G2 conditions. Thirteen such metabolites were identified (C00016: FAD; C00108: Anthranilic acid; C00158: citrate; C00170: 5′-Methylthioadenosine; C00233: 4-Methyl-2-oxopentanoate; C00295: Orotate; C00345: 6-Phospho-D-gluconate; C00500: biliverdin; C01179: 3-(4-Hydroxyphenyl)pyruvate; C01494: Ferulate; C03274: Glycerophosphoglycerol; C08493: Indole-3-carboxaldehyde; and C12246: Protorifamycin I). Compared with the control group, the treatment groups (G1 and G2) showed the same trend, and in some differential metabolites (C00108, C00158, C08493, and C12246), the changes were more pronounced after low-dose treatment (G1). As shown in Figure 5, the boxplot summarizes the trends and extent of changes for these 13 differential metabolites (DMs), with 9 showing clear dose dependence.

**Figure 6:**
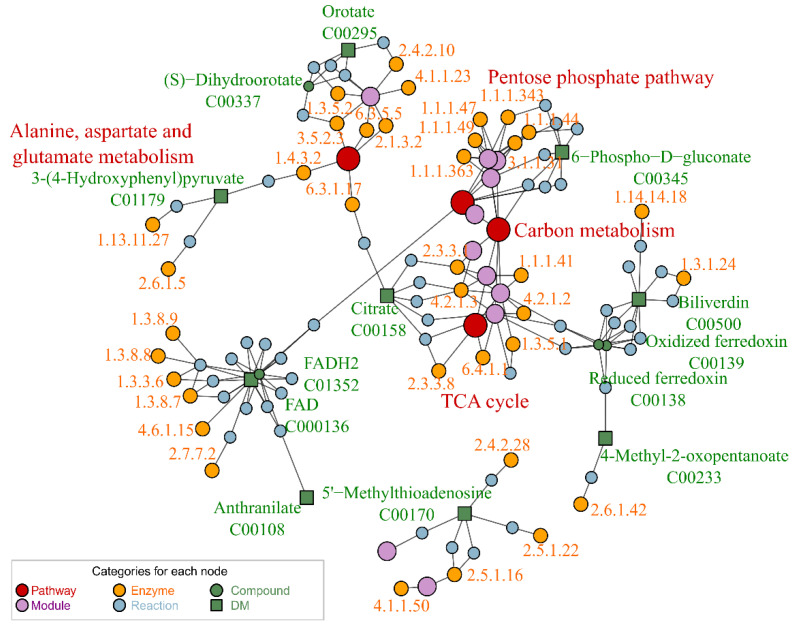
Metabolic network diagram based on FELLA enrichment. The enrichment results based on Fella refer to in Supplementary Materials Table S1.

Subsequently, we integrated the 13 DMs into the FELLA platform to elucidate associated pathways and genomic data (Supplementary Materials Table S1). Our analysis identified that a subset of nine metabolites exhibited significant enrichment within defined metabolic pathways. The interconnections among the KEGG database components, including pathways, modules, enzymes, and reactions, were delineated in Figure 6. These DMs were correlated with key metabolic routes such as the pentose phosphate pathway and the citric acid cycle, as well as the metabolism of alanine, aspartate, glutamate, and carbon metabolism pathways.

### 2.4. Verification of Key Regulatory Genes

**Figure 7:**
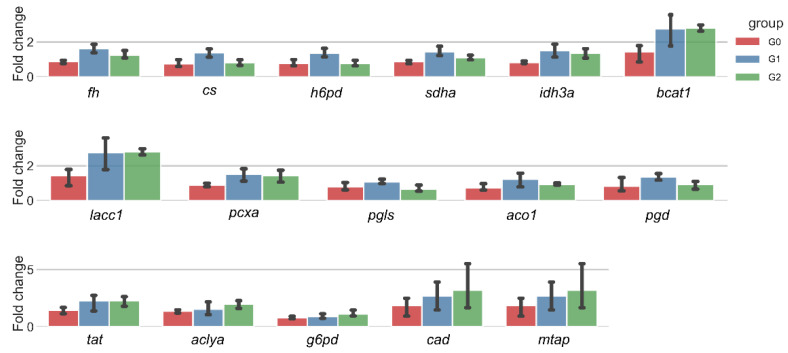
Expression of key genes in enriched pathways, qRT-PCR. β-Actin used as an internal reference gene to normalize. G0, blank group; G1, treatment with a concentration of 4.0 μM; G2, treatment with a concentration of 6.5 μM.

Utilizing the enrichment outcomes, we pinpointed three distinct pathways and identified 16 correlated genes (Figure 7). A comparative quantitative gene expression analysis between the control and experimental groups was conducted employing a one-way ANOVA, uncovering statistically significant variances in the transcription levels of *fh, cs, h6pd, sdha,* and *idh3a* (*p* < 0.05), particularly under the influence of the 4.0 μM treatment. This quintet of genes is implicated in the TCA cycle and the pentose phosphate pathway. Conversely, genes with a direct link to the metabolism of alanine, aspartate, and glutamate, exemplified by *bcat1* and *tat*, manifested an upward trend in expression post-treatment, yet these changes did not reach statistical significance.

### 2.5. Antioxidant Capacity Related to Energy Metabolism

**Figure 8:**
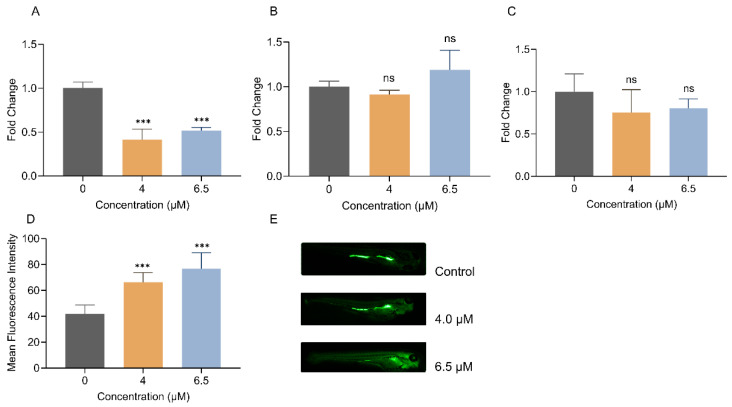
Validation of antioxidant capacity related to energy metabolism. (**A**) GSH activity (*n* = 3). (**B**) MDA level (*n* = 3). (**C**) SOD inhibitory activity (*n* = 3). (**D**) Fluorescence intensity of reactive oxygen species (ROS) in zebrafish larvae (*n* = 15). The blank group was used for normalization, calculating the fold change. (**E**) Accumulation of ROS in zebrafish larvae. (ns, not significant; ***, *p* < 0.001).

The reactive oxygen species (ROS) assay showed that with increasing dosage, the accumulation of ROS in zebrafish gradually increased. Compared to untreated zebrafish larvae, ROS levels in zebrafish increased by 58.8% and 83.5% after exposure to 4.0 μM and 6.5 μM, respectively. Although superoxide dismutase (SOD) activity did not exhibit a dose-dependent pattern, it showed a declining trend after Febrifugin treatment, indicating that Febrifugin can inhibit SOD enzyme activity in zebrafish, affecting their ability to resist oxidative stress. Glutathione (GSH) enzyme activity significantly decreased after exposure to 4.0 μM and 6.5 μM, with the most pronounced inhibitory effect observed at 4.0 μM. Malondialdehyde (MDA) is a cytotoxic substance, and an increase in MDA content can induce lipid peroxidation reactions in the body, causing damage. As shown in Figure 8, although no significant differences were observed, lipid peroxidation reactions were indeed induced after exposure to 4.0 μM and 6.5 μM. In summary, we evaluated whether Febrifugin induces oxidative stress in zebrafish, leading to acute toxicity, based on the relationship between oxidative stress and energy metabolism.

## 3. Discussion

### 3.1. Febrifugin Was a Natural Toxic Component Newly Found in S. macrophylla Seeds

*S. macrophylla* has a long history as a traditional folk medicine and is widely used to treat diseases such as diabetes, malaria, and hypertension. However, despite its extensive use in traditional medicine, its safety and efficacy have not been thoroughly scientifically researched and validated. Consumption of certain parts of *S. macrophylla* may pose health risks, especially if not properly processed or if dosage is not controlled. It is worth noting that risk components in food can pose potential health hazards, particularly natural harmful components in food [13]. In our previous studies, it was found that the popular fruit *Clausena lansium* contains Lansamide alkaloids, toxic components that may cause neurotoxicity and metabolic dysfunction [2] Additionally, plants contain toxins themselves, such as cyanogenic glycosides in almonds, which can cause acute toxicity, such as respiratory depression, if consumed in excess [14]. Previous studies have shown that *S. macrophylla* seeds contain mainly limonoid compounds, which exhibit varying degrees of cytotoxicity to different cells. For instance, swietemicrolides A–D from the bark of *S. macrophylla* did not show significant toxicity to KB and A549 lung cancer cells [15], whereas limonoids derived from the fruit exhibited moderate cytotoxic activity against SW480 and HL-60 cancer cell lines [5]. Current research on limonoid compounds from edible nuts with medicinal value mainly focuses on in vitro toxicity evaluations, and the specific mechanisms of action of individual compounds remain underexplored.

Bioactivity-guided isolation is an efficient separation strategy that facilitates the discovery of compounds with potential properties from different components. Previous studies find that zebrafish behavioral responses have a high consistency with those of mammals in evaluating chemical toxicity. This means that it can effectively enhance the efficiency of the separation process and reduce the preparation of samples for activity assays [16]. In this study, we identified the compound Febrifugin through acute toxicity evaluation in zebrafish larvae. Febrifugin is a mexicanolide-type limonoid compound characterized by a cyclohexane-fused bicyclo [3.3.1]nonane skeleton, with derivatives that feature bridged methyl groups, unique furan rings, alkyl substituents, or high oxidation levels. It exhibits activity in promoting cellular glucose consumption, anti-inflammatory effects, antifungal properties, and inhibiting insect growth and feeding, but its toxicity has not been systematically evaluated [17,18,19]. Limonoid compounds are widely used in agriculture for their insecticidal properties. Mexicanolide-type limonoids exhibit relatively limited activity, mainly focusing on antibacterial and specific cytotoxic effects [20,21]. Mexicanolide-type limonoids isolated from the twigs and leaves of *Cipadessa baccifera* have been evaluated for nematicidal, antifungal, cytotoxic, and acetylcholinesterase inhibitory activities. However, these compounds did not show significant nematicidal and antifungal activities. Compounds such as 3-O-detigloyl-3-O-isobutyryl Febrifugin A, Febrifugin A, Febrifugin, and khaysin T exhibited moderate cytotoxic activity against tested cell lines [22]. Through bioactivity-guided isolation, we found that the toxicity in *S. macrophylla* fruit is due to Febrifugin, which acts as the most representative toxic factor. Using model organisms (zebrafish) with high homology to humans, we obtained results more reliable than cytotoxicity tests to guide the proper consumption. Our study also provided reliable in vivo experimental evidence to further understand the bioactive characteristics of mexicanolide-type limonoids.

### 3.2. The Presence of Chorion Did Not Affect the Production of Developmental Toxicity in Febrifugin

According to our research results, we found that under the same concentration of Febrifugin exposure, the hatching rate of zebrafish embryos was not affected, while the larvae were more sensitive to the toxicity. The chorion on the surface of the embryos acts as a barrier against external chemical stimuli, protecting the development of the zebrafish embryos. Zebrafish have different sensitivities to the environmental pollutant pyraclostrobin at different life stages, in the order of larvae, embryos, and adults [23]. This is consistent with our results. Although there are reports suggesting the removal of the chorion from zebrafish eggs to explore the toxicity mechanisms under chemical exposure [24], we thought that the dechorionation process could cause some damage to the embryo’s health, thus affecting its development. Additionally, after removing the chorion, the exposure of the embryos is different from the actual environmental conditions, which may cause the experimental results that do not fully reflect the toxicity effects under natural conditions.

Morphological assessments of zebrafish are a standard method for evaluating the effects of chemical toxins and environmental contaminants. The incidence of malformations, including pericardial edema and yolk sac enlargement, could be attributed to disruptions in the osmotic balance of the embryos, potentially resulting in pericardial edema. Research indicates that pericardial edema induced by triphenyl phosphate is contingent upon the ionic concentration of the exposure solution; conversely, pyraclostrobin exposure may slow embryonic growth, impairing the heart’s normal operation and possibly inducing cardiac abnormalities and retarded yolk sac resorption [25,26]. Melanin is crucial for zebrafish development. In zebrafish embryos, pigmentation initially emerges in the retinal epithelium, with the *tyr* gene initiating transcription approximately 16.5 hpf. Melanin synthesis in the dorsal lateral skin and retina occurs around the 24 h mark [27]. This synthesis is reliant on tyrosinase activity, which is considered the rate-limiting step [28]. Abnormal skeletal development in zebrafish can be used to confirm the toxic effects of compounds, with detailed and visible skeletal structures forming as early as 5 dpfs [29]. Based on these factors, we investigated whether the chorion serves as a natural barrier affecting the uptake of Febrifugin by evaluating four aspects: deformity rate, body length, melanin deposition, and skeletal development under Febrifugin exposure. Our research results showed that the deformity rate, body length, and melanin deposition of zebrafish began to show significant reduction and inhibition at a treatment concentration of 7.5 μM, with melanin inhibition being the most pronounced at 7.5 μM. This may be due to the specificity of the limiting enzyme in melanin production.

Recent studies have shown that exposure to endocrine-disrupting chemicals such as bisphenol A and norfloxacin can lead to an increase in the craniofacial C-H angle [30], which is consistent with our experimental results. Compared to the MA angle, the C-H angle shows more significant changes at different concentrations. When treated with 20.0 μM, the MA angle showed severe cartilage loss in some larvae, making quantification impossible, indicating severe cartilage development abnormalities in zebrafish larvae. In conclusion, these findings suggest that the chorionic layer in zebrafish embryos exerts no mitigating influence on the toxicity induced by Febrifugin.

### 3.3. The Metabolic Profile of Zebrafish Exposed to Febrifugin Revealed That Acute Toxicity Was Caused by Interference with Energy Metabolism and Oxidative Stress

In this study, we further revealed the mechanism of action of Febrifugin from the perspective of disrupting metabolic functions. Based on FELLA’s enrichment results and differential metabolite analysis, the pathways affected by Febrifugin include the pentose phosphate pathway, the TCA cycle, and alanine, aspartate, and glutamate metabolism. Firstly, we found that Febrifugin significantly affected the expression levels of the key gene *h6pd* in the pentose phosphate pathway. 6-Phospho-D-gluconate (C00345) is produced from glucose-6-phosphate through the catalytic oxidation reaction by the enzyme hexose-6-phosphate dehydrogenase. This reaction not only generates 6-phospho-D-gluconate but also produces NADPH. We found that the content of 6-Phospho-D-gluconate (C00345) significantly increased after treatment, indicating the accumulation of NADPH.

NADPH/NADP^+^ coenzymes provide the driving force for cellular biosynthesis and oxidative defense, and they are produced through the cellular pentose phosphate pathway [31]. A lower NADPH/NADP^+^ ratio may indicate that the cells are experiencing oxidative stress or metabolic disruption. Recent studies have shown that palmitate toxicity can be mediated through this redox reaction, consistent with our observations. Some metabolic enzymes such as isocitrate dehydrogenase-1 have physiological functions of protecting cells from oxidative stress by regulating the intracellular NADP^+^/NADPH ratio [32,33]. Although we cannot directly measure the ratio of NAPH/NADPH^+^ by metabolite content, we still need to use other quantitative methods to confirm the occurrence of oxidative stress disorder caused by Febrifugin.

Furthermore, our investigation identified four genes pivotal in modulating the TCA cycle and energy metabolism. The gene designated as *fh* encodes an enzyme that is primarily responsible for catalyzing the hydration reaction of fumarate, resulting in the production of malate [34]. While there is no immediate correlation between the DMs and *fh*, it is noteworthy that fumarate and malate serve as essential intermediates within the TCA cycle. The *cs* gene encodes for citrate synthase, an enzyme that facilitates the condensation of acetyl-CoA with oxaloacetate to initiate the TCA cycle by generating citrate [35]. The gene *cs* is directly linked to the differential metabolite C00158 (citrate). The citrate synthesized by citrate synthase undergoes conversion to isocitrate within the citric acid cycle, with a concurrent escalation in the metabolite’s concentration during the metabolic process. The *Sdha* gene is implicated in the oxidative conversion of succinate to fumarate, concurrently transferring electrons to ubiquinone, thus playing a dual role in both the TCA cycle and the electron transport chain [36]. *Idh3a* regulates the formation of isocitrate dehydrogenase which catalyzes the decarboxylation of isocitrate to α-ketoglutarate, accompanied by the reduction of NAD^+^ to NADH, representing a key step in the TCA cycle [36]. Although DMs (C00016, FAD) are not direct participants, FAD serves as an electron carrier in other steps of the TCA cycle, such as in the succinate dehydrogenase (SDH) reaction. In conclusion, the enzymes encoded by these genes play roles at different points in the metabolic pathways, directly or indirectly relating to the differential metabolites through various metabolic processes. The citric acid cycle, glycolysis, alanine, aspartate, and glutamate metabolism are the primary related pathways. An imbalance in the redox state can also severely disrupt the metabolic pathways of alanine, aspartate, and glutamate [37]. Although we did not detect a direct link between compound treatment and this metabolic pathway, the enrichment results might be due to the disruption of energy metabolism. Other DMs, such as C00108 (Anthranilic acid), are considered a potential biomarker in the tryptophan metabolic pathway associated with physiological diseases and metabolic disorders; C00500, biliverdin is regarded as a cytotoxic metabolic waste, and its accumulation in zebrafish after treatment may also be a cause of toxicity [38,39].

In addition to verifying gene expression, we also assessed the oxidative stress condition to confirm that the cause of acute toxicity was the disruption of the energy cycle pathway by measuring the activity of antioxidant enzymes related to energy metabolism. We also verified the occurrence of oxidative stress induced by Febrifugin by evaluating ROS accumulation. Compared to the control group, ROS levels significantly increased under Febrifugin treatment. Excessive ROS accumulation, as a key initiator of oxidative stress, can have a series of adverse effects on the body [40]. GSH peroxidase helps eliminate various forms of ROS and RNS, including peroxynitrite, maintaining the balance of redox reactions within the cell. GSH serves as a protective shield against the proliferation of ROS within cellular compartments. MDA represents the terminal product of polyunsaturated fatty acid peroxidation in cellular membranes [41], thereby providing an index of lipid peroxidation levels and serving as an indirect biomarker for cellular injury. SOD, a pivotal scavenger of oxygen-derived free radicals, is essential for the preservation of metabolic equilibrium [42]. In this study, under Febrifugin exposure conditions, we found that ROS levels increased significantly, MDA levels did not show significant differences but showed an increasing trend, and the inhibition of GSH activity was more pronounced than that of SOD. These phenomena are consistent with the oxidative stress induced by various compound toxicities. Notably, in the inhibition of GSH and SOD activities, a higher inhibition effect was observed at 4.0 μM compared to the higher concentration of 6.5 μM; we speculate that this may be due to feedback regulation mechanisms in zebrafish larvae adapting to environmental toxic disturbances, but this phenomenon was not observed in ROS levels.

## 4. Materials and Methods

### 4.1. Chemicals and Materials

The *S.macrophylla* seeds were procured from the herbal marketplace in Chengdu, located in Sichuan province, China. Dimethyl sulfoxide (DMSO), Alcian blue, and 2′,7′-Dichlorodihydrofluorescein diacetate were sourced from Solarbio Life Sciences Co. Ltd., Beijing, China. Ultra-performance liquid chromatography (UPLC)-grade solvents were acquired from Thermo Fisher Scientific Inc., Waltham, MA, USA. Adult zebrafish were obtained from Nanjing Ezerinka Co. Ltd., Nanjing, China.

### 4.2. The Process of Isolating and Characterizing the Principal Constituents

The isolation of the major toxic compounds was guided by bioactivity. After drying and pulverizing 700 g of *S. macrophylla* fruits, they were soaked in 3L of 80% methanol for 48 h with regular stirring. The mixture was then filtered and concentrated using a rotary evaporator. This process was repeated twice, yielding 47.6 g of crude extract. The extract was resuspended in water and subjected to extraction with petroleum ether, dichloromethane, and ethyl acetate, resulting in four fractions. The FrB fraction was further subjected to column chromatography using dichloromethane–methanol as the mobile phase. Increasing polarity resulted in eight fractions (Fr B.1 ~ Fr B.8). Fractions Fr B.1 (19.2 g), Fr B.2 (2.8 g), and Fr B.3 (0.45 g) were combined and further purified using a MeOH-H_2_O solvent system. Subsequent preparative HPLC with MeCN-H_2_O as the mobile phase under isocratic conditions yielded seven fractions. The objective compounds were successfully isolated, and their molecular architectures were elucidated through ^1^H and ^13^C NMR spectroscopy, complemented by high-resolution (HR) ESI-MS (found *m*/*z* 553.2779, calculated as [M+H]^+^ 553.27959). The spectral data are presented in the Supplementary Materials section (Figure S1).

### 4.3. Evaluation of Febrifugin Content in Different Parts of S. macrophylla

Nut kernels and peel of *S. macrophylla* (0.5 g) were separately weighed out and extracted with 90% MeOH using ultrasonication at a constant material-to-liquid ratio (1:50, *w*/*v*) at 40 °C for 90 min. Control of solution mass before and after extraction was conducted to minimize errors due to solvent evaporation. The extracts were filtered and quantitatively analyzed using LC-MS/MS, with reference to a Febrifugin standard solution.

### 4.4. Acute Toxicity Assessment

The zebrafish broodstock maintenance and spawning procedures adhered to the protocols we have previously detailed [2]. At 3 dpfs, a sample of sixty zebrafish larvae was selected for toxicity assessment. All experimental protocols were in accordance with the ‘Guidelines for the Care and Use of Laboratory Animals in China’, and the study was granted approval by the Institutional Animal Care and Use Committee of Northwest A&F University (Permit Number: XN2024-0403, dated 23 July 2023). Each experimental condition was conducted in triplicate, with each replicate consisting of 20 larvae. The larvae were allocated into 6-well plates, housing 20 larvae per well, and subjected to varying concentrations of Febrifugin (3.0 mL at 2.5, 5.0, 7.5, 10.0, and 20.0 μM). A 0.1% (*v*/*v*) DMSO treatment served as the control. The E3 medium was replenished every 24 h, and after 48 h, the mortality of the zebrafish larvae was documented. The lethality rate was ascertained by expressing the number of deceased larvae as a percentage of the initial count per replicate. Following statistical evaluation, the LC_10_ concentrations, identified as 4.0 μM and 6.5 μM, were chosen for further mechanistic studies, encompassing metabolic profiling (Section 4.8), oxidative stress assessment (Section 4.9 and Section 4.10), and the verification of pivotal regulatory genes (Section 4.11).

### 4.5. Developmental Toxicity Assessment

The embryonic toxicity of Febrifugin on zebrafish was evaluated following a methodology akin to prior research [2]. Specifically, non-viable eggs were promptly discarded within the initial 24 hpf. Each group selected 60 normal zebrafish embryos for developmental toxicity evaluation, with 20 embryos exposed to different concentrations of Febrifugin solution, while the control group was exposed to 0.1% (*v*/*v*) DMSO. The culture medium E3 was updated daily, and the incidence of larval developmental abnormalities was monitored until 72 hpf. Zebrafish larvae with developmental abnormalities were placed under the Nikon SMZ25 stereomicroscope (Nikon Corporation, Tokyo, Japan), and ImageJ (version 1.54) was used to determine their abnormal state. Each experimental concentration has three independent biological replicates.

### 4.6. Determination of Melanin Content

For the determination of melanin in zebrafish larvae, the treatment method was the same as for developmental toxicity, and the method was based on previous research with some modifications [43]. The melanin assessment method involved measuring the absorbance of melanin at 490 nm to determine its content. Every 30 zebrafish were homogenized in PBS and centrifuged, and the pellet was dissolved in 1M NaOH, then heated at 95 °C for 30 min until fully dissolved. After centrifugation, the absorbance of the supernatant was measured at 490 nm. Three biological replicates were set up for subsequent differential analysis.

### 4.7. Skeletal Development Status

Alcian blue staining is commonly used in zebrafish studies to detect and analyze skeletal, cartilage, and other tissue structures containing acidic polysaccharides. Based on previous studies, the following protocol was modified [44]: zebrafish were exposed to different concentrations of Febrifugin solution (2.5, 5.0, 7.5, 10.0, and 20.0 μM) after 24 hpf, while the blank control group was exposed to E3 solution containing 0.1% DMSO. After 48 h, zebrafish larvae were fixed in 4% paraformaldehyde and then bleached with a solution of 3% hydrogen peroxide and 0.5% KOH. Next, the larvae were incubated overnight in 0.1% acidic Alcian blue ethanol solution. Post-staining, the larvae were resuspended in acidic ethanol (5% concentrated hydrochloric acid and 70% ethanol) and gradually rehydrated through different concentrations of acidic ethanol (75%, 50%, 25%, and 0%). The specimens were preserved in glycerol. Observations were made using a stereomicroscope (Nikon Corporation, Tokyo, Japan), and the results were used to measure the ceratohyal bone angles and Meckel’s cartilage angle.

### 4.8. Preparation of Metabolome Samples, LC-MS/MS Data Collection and Processing

At 3 dpfs, zebrafish larvae were categorized into three experimental groups. The control group was maintained in an E3 medium supplemented with 0.1% DMSO, whereas the experimental groups were subjected to Febrifugin at concentrations of 4.0 μM and 6.5 μM. Upon reaching 5 dpfs, the larvae were harvested following dual rinses with deionized water and promptly cryopreserved in liquid nitrogen. Sample homogenization was conducted using a JY92-IIDN cell disruptor (Ningbo Scientz Biotechnology Co., Ltd., Ningbo, China), operated at 20% power for a duration of 3 min with a pulsing cycle of 1 s on and 3 s off. Throughout homogenization, samples were kept in an ice bath to preserve integrity. Post-homogenization, the samples underwent centrifugation at 12,000 revolutions per minute (rpms) at 4 °C, after which the supernatant was extracted for subsequent metabolomic profiling. Metabolite profiling was performed on a Thermo Q Exactive Focus mass spectrometer equipped with a 2.6 μm Accucore AQ C_18_ column (150 × 2.1 mm), both from Thermo Fisher Scientific, Waltham, MA, USA. The analytical procedure adhered to the data-dependent MS2 (dd-MS2) methodology as previously delineated in our publications [45]. For detailed mass spectrometry acquisition parameters and LC-MS/MS data processing protocols, readers can be directed to our prior work.

### 4.9. Determination of SOD, GSH Enzyme Activity, and MDA Content

We assessed the impact of Febrifugin exposure on the zebrafish’s antioxidant defenses by quantifying SOD and GSH enzyme activities, as well as MDA levels. The experimental setup for the zebrafish larvae paralleled that utilized in the metabolomics analysis. Before conducting the assays, the concentration of total protein was ascertained using a BCA protein assay kit to standardize the data (Solarbio Biotechnology Co., Ltd., Beijing, China). Homogenization of all zebrafish larvae was carried out in phosphate-buffered saline (PBS), and the resulting supernatant was reserved for subsequent biochemical analysis. The biochemical assays were executed with a microplate reader (Synergy HLx, Gene Company Limited, Hong Kong, China), employing various absorbance readings in accordance with the manufacturer’s guidelines for the respective assay kits. SOD and GSH activities (Beyotime Biotechnology Co., Ltd., Shanghai, China), as well as MDA content levels (Solarbio Biotechnology Co., Ltd., Beijing, China), were measured using a reagent kit.

### 4.10. ROS Accumulation

To ascertain the spatial distribution of ROS, the embryos underwent a dual rinse with embryo water subsequent to exposure, followed by incubation in the dark at 28°C with 2′,7′-dichlorodihydrofluorescein diacetate (H2DCF-DA) at a concentration of 20 μg/mL for a duration of 1 h. Post-staining, the embryos were subjected to anesthesia using 0.02% tricaine. Fluorescence imaging was conducted with a Nikon SMZ25 stereomicroscope equipped for fluorescence, provided by Nikon Corporation, Tokyo, Japan. The quantification of fluorescence intensity was achieved by employing Image J software (version 1.54).

### 4.11. qRT-PCR Assay

Zebrafish larvae at 3 dpfs were subjected to Febrifugin at concentrations of 0, 4.0, and 6.5 μM, aligning with the protocols established for metabolomic analysis. Total RNA extraction from each zebrafish embryo group (*n* = 100) was facilitated using AG RNAex Pro Reagent, procured from Accurate biology Co., Ltd., Changsha, China, following the manufacturer’s guidelines. RNA integrity and purity were subsequently assessed via a spectrophotometer from Thermo Fisher Scientific. Reverse transcription of the RNA was carried out with the Evo M-MLV RT kit (Accurate biology Co., Ltd., Changsha, China), which includes a gDNA removal option for qPCR II. The RT-PCR reactions were executed on a CFX 96 Touch Real-Time PCR Detection System (Bio-Rad Laboratories, Inc., Hercules, CA, USA), strictly adhering to the manufacturer’s kit protocol. Quantitative expression levels were determined employing the 2^−∆∆Ct^ method, and β-actin was used as an internal reference gene to normalize the sequences of the primers utilized for qPCR. The Primers used for qPCR analysis refered to Supplementary Materials Table S2 and the results were detailed in the Supplementary Materials Table S3.

### 4.12. Data Analysis

All data visualization and statistical analyses were conducted utilizing the ggplot2 package within the R (version 4.4.0). For the purpose of group comparison, Student’s *t*-test was employed to evaluate the statistical difference between the two groups. When assessing the variance among multiple groups, a one-way analysis of variance (ANOVA) was applied. The threshold for statistical significance was set at a *p*-value of less than 0.05.

## 5. Conclusions

In this study, we employed activity-guided isolation to extract a representative toxic compound, Febrifugin, from *S. macrophylla*. Febrifugin, a mexicanolide-type limonoid, was systematically evaluated for its toxicological effects on zebrafish. We found that zebrafish larvae exhibited greater sensitivity to Febrifugin toxicity compared to zebrafish embryos at the same concentration. However, the presence of the chorion in zebrafish embryos did not affect the manifestation of Febrifugin’s toxic effects. We revealed that Febrifugin directly impacts the *h6pd*-mediated pentose phosphate pathway, leading to metabolic disruption. Febrifugin also interferes with the TCA cycle and energy metabolism. Furthermore, we confirmed that Febrifugin induces oxidative stress, resulting in toxicity, by measuring reactive oxygen species accumulation, the activity of antioxidant enzymes (such as SOD and GSH), and the levels of lipid peroxides (MDA). Additionally, we propose that zebrafish larvae may possess feedback regulatory mechanisms to maintain homeostasis and adapt to the adverse effects of environmental toxicity, although this hypothesis requires further validation. Our research enriches the diversity of activities associated with mexicanolide-type limonoids. It further enhances the understanding of the toxicity of the medicinal plant *S. macrophylla*, providing theoretical support for its rational dietary application.

## Data Availability

Data are contained within the article.

## Supplementary Materials

The following supporting information can be downloaded at https://www.mdpi.com/article/10.3390/ijms25179753/s1.
