# Supplementary material for: Toxicity and Metabolomic Dysfunction Invoked by Febrifugin, a Harmful Component of Edible Nut of Swietenia macrophylla
Source: Int J Mol Sci. 2024 Sep 9;25(17):9753. doi: 10.3390/ijms25179753 (PMC11396426; doi:10.3390/ijms25179753)
Supplement: Supplementary file 1 [file ijms-25-09753-s001.zip › ijms-3170504-supplementary.pdf]

Supplementary materials

Toxcity and Metabolomic Dysfuction Invoked by Febrifugin, a Harmful Component of Edible Nut of *Swietenia macrophylla*

Xiaoyue Zhang [1], Qinyang Song [1], Hanghang Zheng [2], Rui Wang [2], and Qiang Zhang [1,*]

[1] Shaanxi Key Laboratory of Natural Products & Chemical Biology, College of Chemistry & Pharmacy, Northwest A&F University, Yangling 712100, China.
[2] National Narcotics Laboratory Shaanxi Regional Center, Narcotics Technical Center of Shaanxi Provincial Public Security Department, Key Laboratory of Drugs analysis & intelligent-Monitoring, Xi'an, 712046, China
* Correspondence: zhangq@nwsuaf.edu.cn

*Corresponding author, Qiang Zhang, Email: zhangq@nwsuaf.edu.cn

Contents

Figure S1. ^{1}H (400 MHz) ,^{13}C (100 MHz) NMR and LC-MS/MS spectrum of Febrifugin.

Table S1. Metabolic enrichment results based on Fella ($p < 0.01$)

Table S2. Primers used for qPCR analysis

Table S3. qPCR analysis results.

Figure S1. ^{1}H (400 MHz) ,^{13}C (100 MHz) NMR and LC-MS/MS spectrum of Febrifugin.

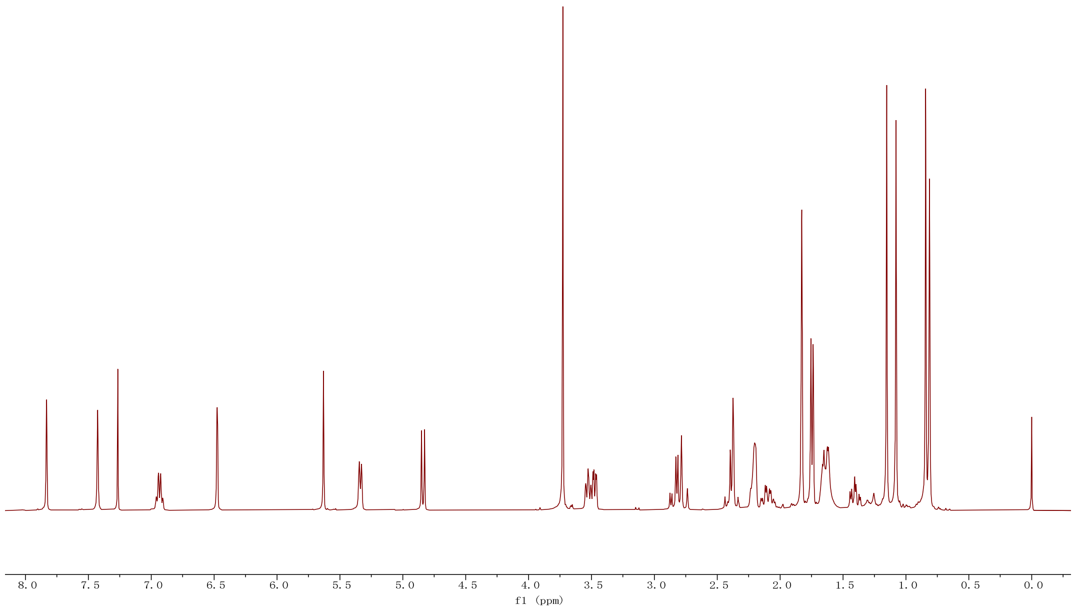

^{1}H NMR (400 MHz, $CDCl_3$) δ 0.80-0.90,(m,2H),1.05-1.5(m,3H),1.53-1.82(m,4H),2.10-2.40(m,4H),2.75-2.82(m,3H),3.48-3.75(m,3H),4.85(d,1H),5.35(m,1H),5.65(s,1H),6.47(s,1H),6.95(m,1H),7.42-7.83(m,2H).

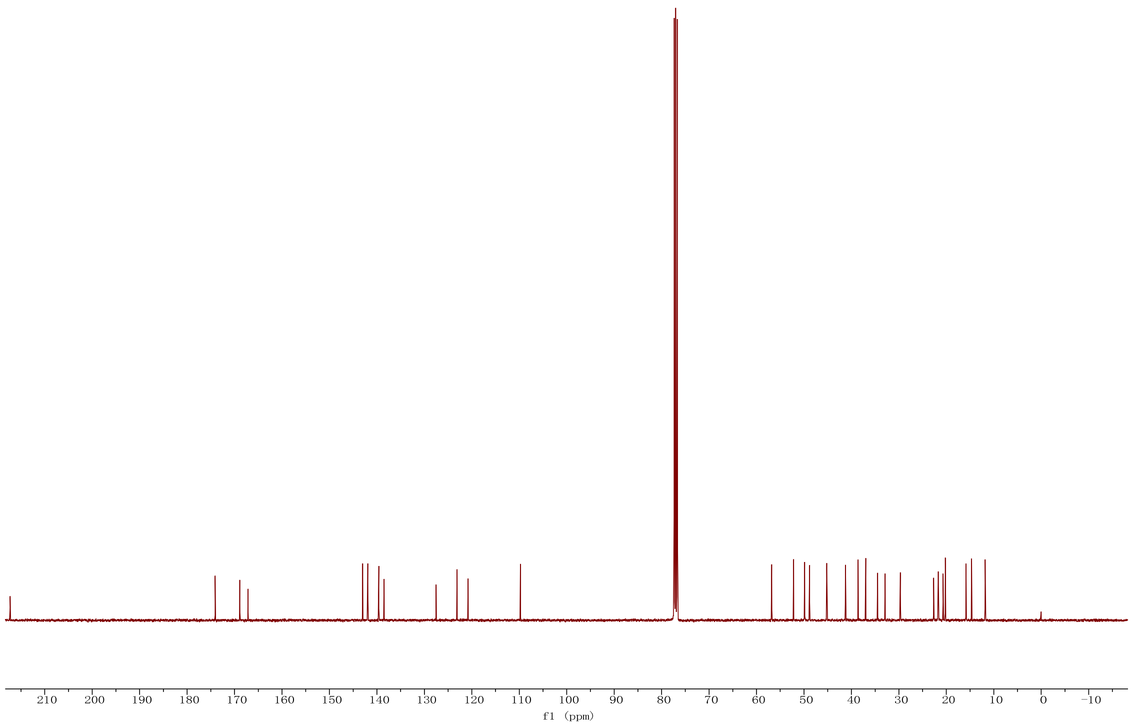

^{13}C NMR (101 MHz, $CDCl_3$) δ 217.30, 174.08, 168.89, 167.14, 143.00, 141.91, 139.60, 138.49, 127.53, 123.10, 120.79,

109.74, 77.35, 77.08, 77.03, 76.71, 76.64, 56.80, 52.19, 49.85, 48.82, 45.19, 41.26, 38.61, 36.98, 34.49, 32.91, 29.69, 22.66, 21.70, 20.67, 20.22, 15.85, 14.68, 11.82, 0.00.

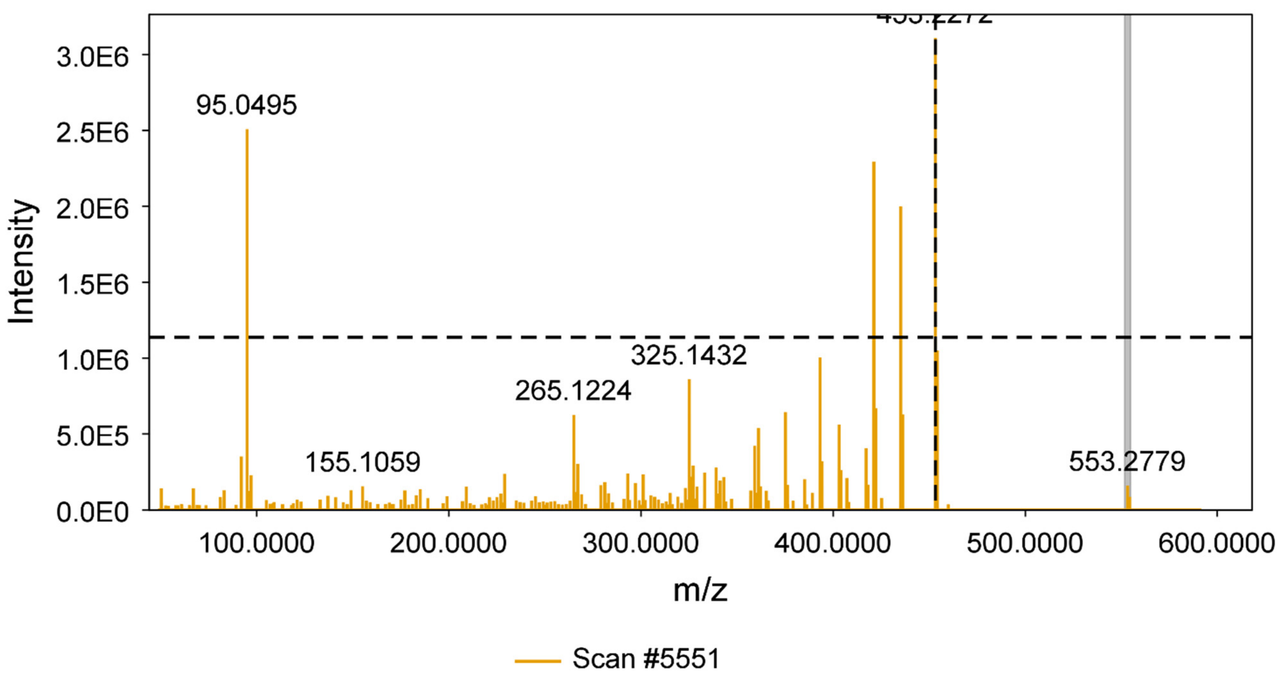

Found m/z 553.2779

Table S1. Metabolic enrichment results based on Fella (*p* < 0.01)

KEGG.id	Entry.type	KEGG.name	p.score
dre00030	pathway	Pentose phosphate pathway - Danio rerio (zebr...	0.000123
dre00020	pathway	Citrate cycle (TCA cycle) - Danio rerio (zebr...	0.000140
dre00250	pathway	Alanine, aspartate and glutamate metabolism -...	0.007235
dre01200	pathway	Carbon metabolism - Danio rerio (zebrafish)	0.012266
M00004	module	Pentose phosphate pathway (Pentose phosphate ...	0.000001
M00006	module	Pentose phosphate pathway, oxidative phase, g...	0.000001
M00008	module	Entner-Doudoroff pathway, glucose-6P => glyce...	0.000001
M00051	module	De novo pyrimidine biosynthesis, glutamine (+...	0.000001
M00010	module	Citrate cycle, first carbon oxidation, oxaloa...	0.000002
M00173	module	Reductive citrate cycle (Arnon-Buchanan cycle...	0.000003
M00012	module	Glyoxylate cycle	0.000118
M00368	module	Ethylene biosynthesis, methionine => ethylene	0.000403

M00009	module	Citrate cycle (TCA cycle, Krebs cycle)	0.000451
M00133	module	Polyamine biosynthesis, arginine => agmatine ...	0.000985
M00005	module	PRPP biosynthesis, ribose 5P => PRPP	0.001915
M00171	module	C4-dicarboxylic acid cycle, NAD - malic enzym...	0.009360
1.1.1.343	enzyme	phosphogluconate dehydrogenase (NAD+-dependen...	0.000001
1.1.1.44	enzyme	phosphogluconate dehydrogenase (NADP+-depende...	0.000001
1.3.1.24	enzyme	biliverdin reductase	0.000001
1.3.5.2	enzyme	dihydroorotate dehydrogenase (quinone)	0.000001
2.3.3.1	enzyme	citrate (Si)-synthase	0.000001
2.3.3.8	enzyme	ATP citrate synthase	0.000001
2.4.2.10	enzyme	orotate phosphoribosyltransferase	0.000001
3.1.1.31	enzyme	6-phosphogluconolactonase	0.000001
3.5.2.3	enzyme	dihydroorotase	0.000001
4.2.1.3	enzyme	aconitate hydratase	0.000001
4.6.1.15	enzyme	FAD-AMP lyase (cyclizing)	0.000001
6.3.5.5	enzyme	carbamoyl-phosphate synthase (glutamine-hydro...	0.000001
2.5.1.16	enzyme	spermidine synthase	0.000001
1.14.14.18	enzyme	heme oxygenase (biliverdin-producing)	0.000001
2.6.1.42	enzyme	branched-chain-amino-acid transaminase	0.000003
2.5.1.22	enzyme	spermine synthase	0.000004
2.1.3.2	enzyme	aspartate carbamoyltransferase	0.000005
2.4.2.28	enzyme	S-methyl-5'-thioadenosine phosphorylase	0.000008
2.6.1.5	enzyme	tyrosine transaminase	0.000008
4.1.1.23	enzyme	orotidine-5'-phosphate decarboxylase	0.000133
1.1.1.49	enzyme	glucose-6-phosphate dehydrogenase (NADP+)	0.000213
6.4.1.1	enzyme	dan'shing'f	0.000343
1.1.1.41	enzyme	isocitrate dehydrogenase (NAD+)	0.000977
1.13.11.27	enzyme	4-hydroxyphenylpyruvate dioxygenase	0.002373
1.1.1.363	enzyme	glucose-6-phosphate dehydrogenase [NAD(P)+]	0.002633
1.3.8.7	enzyme	medium-chain acyl-CoA dehydrogenase	0.002668

4.1.1.50	enzyme	adenosylmethionine decarboxylase	0.003894
1.1.1.47	enzyme	glucose 1-dehydrogenase [NAD(P)+]	0.004008
1.3.8.8	enzyme	long-chain acyl-CoA dehydrogenase	0.004734
4.2.1.2	enzyme	fumarate hydratase	0.005061
6.3.1.17	enzyme	beta-citrylglutamate synthase	0.005553
1.3.8.9	enzyme	very-long-chain acyl-CoA dehydrogenase	0.005802
2.7.7.2	enzyme	FAD synthase	0.008679
1.3.3.6	enzyme	acyl-CoA oxidase	0.008960
1.3.5.1	enzyme	succinate dehydrogenase	0.009660
C00016	compound	FAD	0.000001
C00108	compound	Anthranilate	0.000001
C00158	compound	Citrate	0.000001
C00170	compound	5'-Methylthioadenosine	0.000001
C00233	compound	4-Methyl-2-oxopentanoate	0.000001
C00295	compound	Orotate	0.000001
C00345	compound	6-Phospho-D-gluconate	0.000001
C00500	compound	Biliverdin	0.000001
C01179	compound	3-(4-Hydroxyphenyl)pyruvate	0.000001
C01494	compound	Ferulate	0.000001
C00337	compound	(S)-Dihydroorotate	0.000012
C01352	compound	FADH2	0.000256

Table S2. Primers used for qPCR analysis

**Gene Symbol**	**Oligo Name**	**Sequence (5' to 3')**
pgd	pgd-F	CAAGGACGCAGACGGCACTAAC
	pgd-R	GACGAGAGGCATCTGGCAAAGAC
pgls	pgls-F	GCCGAGGACTATGCCAGCAAAC
	pgls-R	TCCCATTCCCAGCAACAAGACATC
h6pd	h6pd-F	CACGGCAACCTCCAATACCCTTC
	h6pd-R	CTTCCAGTCACCGCTGCTCAC
cs	cs-F	CGTGAAGGCAGCAGCATCGG
	cs-R	CTGTGAACTGTGGCTCGGTGTATC

bcat1	bcat1-F	AGAACTTGCTCGCCAATGGAGTG
	bcat1-R	CTGCCGACTGGACTGACAACAC
cad	cad -F	GACGATGCTGCCTCTGCTGTTC
	cad -R	CTCCTGCTCCAAATCCACCTCAAC
lacc1	lacc1-F	TGATGTGTGGGTGATGGGCAAAC
	lacc1-R	CAGGGTCAGTGAAGAGCAATGGC
mtap	mtap-F	GGAGGGAAGGACAGAGCGGTATG
	mtap-R	TTGGCATGATGGTGTGTTGTCTCC
tat	tat-F	CAGCGTGTTGTGTAACCCAGGAG
	tat-R	CAGGACTTCTCAGGCAGCAGATTG
aclya	aclya-F	GCTCTGGATGCCGCTGCTAAG
	aclya-R	CCTGTGCCCGATGCCCATAATG
aco1	aco1-F	GCCCTAAACGACCACATGACAGAG
	aco1-R	AACTGGAACGGCACTTGGACATC
g6pd	g6pd-F	TGTAAAGAACGAACGCTGGGATGG
	g6pd-R	GCAGACGCACCTCCGCTTTC
pcxa	pcxa-F	CTTCACATCAAGGCTCTGGCTCTG
	pcxa-R	TTCAGGGCTTTAGGGTGGAAATGC
idh3a	idh3a-F	GGATTGATTGGCGGTCTTGGAGTC
	idh3a-R	TCATGACGGCACTAAGCAAAAGGG
fh	fh-F	CGGAGGAGTCACGGAGAGGATG
	fh-R	ATGATGGCGTCTGCGATCTTTGG
sdha	sdha-F	GTGCCTGTGCCCTGACCATTG
	sdha-R	TTAGCGACCGATGCCTCTCCTG
β-actin	β-actin-F	AACGAACGACCAACCTAAACCTCTC
	β-actin-R	CTCCCTTTCCAGTTTCCGCATCC

Table S3. qPCR analysis results.

gene symbol	Group	Fold change
aclya	G0	1.424667252
aclya	G0	1.210518393
aclya	G0	1.480028544
aclya	G1	2.181015465
aclya	G1	1.399585866
aclya	G1	1.060687741
aclya	G2	1.602139755

aclya	G2	2.281527432
aclya	G2	2.117702602
aco1	G0	0.974764084
aco1	G0	0.624435732
aco1	G0	0.610526152
aco1	G1	1.326385371
aco1	G1	1.57734692
aco1	G1	0.78867346
aco1	G2	0.891928519
aco1	G2	0.896577376
aco1	G2	1.012203943
bcat1	G0	1.79082579
bcat1	G0	0.854483773
bcat1	G0	1.708967546
bcat1	G1	2.882858447
bcat1	G1	3.60500185
bcat1	G1	1.783857039
bcat1	G2	2.620786808
bcat1	G2	2.808889751
bcat1	G2	2.979354926
cad	G0	2.497742928
cad	G0	0.935056211
cad	G0	2.208589046
cad	G1	3.910898245
cad	G1	2.775023521
cad	G1	1.474269217
cad	G2	1.670175839
cad	G2	2.449521577
cad	G2	5.511710068
cs	G0	0.988371284

cs	G0	0.595892915
cs	G0	0.689262294
cs	G1	1.431472545
cs	G1	1.602139755
cs	G1	1.128964405
cs	G2	0.840896415
cs	G2	0.981118975
cs	G2	0.656333056
fh	G0	0.951400705
fh	G0	0.767439539
fh	G0	0.925384603
fh	G1	1.610490332
fh	G1	1.869302432
fh	G1	1.385109468
fh	G2	1.084853325
fh	G2	1.513092304
fh	G2	1.189207115
g6pd	G0	0.917798911
g6pd	G0	0.701614272
g6pd	G0	0.78935709
g6pd	G1	1.141258021
g6pd	G1	0.815425512
g6pd	G1	0.740014272
g6pd	G2	0.973076409
g6pd	G2	0.958018221
g6pd	G2	1.457125359
h6pd	G0	0.992232419
h6pd	G0	0.721339334
h6pd	G0	0.638938952
h6pd	G1	1.313234904

h6pd	G1	1.636512929
h6pd	G1	1.147206418
h6pd	G2	0.635350676
h6pd	G2	0.954703729
h6pd	G2	0.712333626
idh3a	G0	0.918594468
idh3a	G0	0.771105413
idh3a	G0	0.809442217
idh3a	G1	1.512436949
idh3a	G1	1.874979731
idh3a	G1	1.132393207
idh3a	G2	1.078760798
idh3a	G2	1.391725803
idh3a	G2	1.618183257
lacc1	G0	1.79082579
lacc1	G0	0.854483773
lacc1	G0	1.708967546
lacc1	G1	2.882858447
lacc1	G1	3.60500185
lacc1	G1	1.783857039
lacc1	G2	2.620786808
lacc1	G2	2.808889751
lacc1	G2	2.979354926
mtap	G0	2.497742928
mtap	G0	0.935056211
mtap	G0	2.208589046
mtap	G1	3.910898245
mtap	G1	2.775023521
mtap	G1	1.474269217
mtap	G2	1.670175839

mtap	G2	2.449521577
mtap	G2	5.511710068
pcxa	G0	1
pcxa	G0	0.86154616
pcxa	G0	0.806641759
pcxa	G1	1.623801182
pcxa	G1	1.830039769
pcxa	G1	1.124571196
pcxa	G2	1.060228333
pcxa	G2	1.544216464
pcxa	G2	1.749417948
pgd	G0	1.325236643
pgd	G0	0.563016848
pgd	G0	0.623624711
pgd	G1	1.378524588
pgd	G1	1.553610655
pgd	G1	1.17945878
pgd	G2	0.656617452
pgd	G2	1.059310113
pgd	G2	1.100473954
pgls	G0	1.040210135
pgls	G0	0.615305588
pgls	G0	0.741940295
pgls	G1	1.013081331
pgls	G1	1.23648951
pgls	G1	0.976877794
pgls	G2	0.582871594
pgls	G2	0.543838427
pgls	G2	0.892701651
sdha	G0	0.945647887

sdha	G0	0.758843875
sdha	G0	0.927793086
sdha	G1	1.374350518
sdha	G1	1.757774496
sdha	G1	1.221581399
sdha	G2	1.10860852
sdha	G2	1.242934266
sdha	G2	0.978572062
tat	G0	1.575298251
tat	G0	1.145220182
tat	G0	1.688362861
tat	G1	2.7463205
tat	G1	2.67585511
tat	G1	1.385109468
tat	G2	1.796264746
tat	G2	2.432601496
tat	G2	2.634446716
